# Short-term virus-host interactions and functional dynamics in recently deglaciated Antarctic tundra soils

**DOI:** 10.1093/ismeco/ycaf157

**Published:** 2025-09-09

**Authors:** Esther Rubio-Portillo, Rebeca Arias-Real, Esther Rodríguez-Pérez, Lluis Bañeras, Josefa Antón, Asunción de los Ríos

**Affiliations:** Department of Physiology, Genetics and Microbiology, University of Alicante, 03690-Alicante, Spain; Department of Biogeochemistry and Microbial Ecology, Museo Nacional de Ciencias Naturales-CSIC, José Gutiérrez Abascal 2, 28006-Madrid, Spain; Department of Biogeochemistry and Microbial Ecology, Museo Nacional de Ciencias Naturales-CSIC, José Gutiérrez Abascal 2, 28006-Madrid, Spain; Group of Molecular Microbial Ecology, Institute of Aquatic Ecology, University of Girona, 17004-Girona, Spain; Department of Physiology, Genetics and Microbiology, University of Alicante, 03690-Alicante, Spain; Department of Biogeochemistry and Microbial Ecology, Museo Nacional de Ciencias Naturales-CSIC, José Gutiérrez Abascal 2, 28006-Madrid, Spain

**Keywords:** Antarctica, biodiversity, glacier forefield, climate change, biotic interactions, virus-host interactions, Pseudomonas phages

## Abstract

Long-term chronosequence studies have shown that, as glaciers retreat, newly exposed soils become colonized through primary succession. To determine the key drivers of this process and their vulnerability to climate change, the short-term responses of these pioneering microbial communities also need to be elucidated. Here, we investigated how the taxonomic and functional structure of microbial communities, including viruses, changed over a 7-year period in an Antarctic glacier forefield. Using metagenomics and metatranscriptomics we assessed the influence of both abiotic and biotic factors on these communities. Our results revealed a highly heterogeneous bacteria-dominated microbial community, with *Pseudomonas* as the most abundant genus, followed by *Lysobacter*, *Devosia*, *Cellulomonas*, and *Brevundimonas*. This community exhibited the capacity for aerobic anoxygenic phototrophy, carbon and nitrogen fixation, and sulfur cycling, processes vital for survival in nutrient-poor environments. 52 high-quality metagenome-assembled genomes (MAGs) were recovered, representing both transient and cosmopolitan taxa, some of which were able to rapidly respond to environmental changes. A diverse and highly dynamic collection of lytic and temperate viruses was identified across all samples, with high clonal viral genomes typically detected in only one of the eight samples analyzed. Metatranscriptomic analyses confirmed the activity of lytic viruses, while prophage genomes featured much lower expression levels. Prophages appeared to influence host fitness through the expression of genes encoding membrane transporters. Additionally, the abundance of genes linked to antimicrobial compound synthesis and resistance, along with antiphage defense systems, highlights the importance of biotic interactions in driving microbial community succession and shaping short-term responses to environmental fluctuations.

## Introduction

Antarctic tundra ecosystems are confined to limited ice-free coastal zones, which, despite covering < 0.4% of the continent, host its richest terrestrial biodiversity [1]. They develop through primary succession in areas newly exposed after prolonged glacial retreat and are dominated by nonvascular vegetation [2]. Microbial communities are vital to these ecosystems [3, 4], but their assembly and functional dynamics are still poorly understood, particularly in relation to natural ecological processes and the impacts of regional climate change and ice retreat.

Glaciers lost an average of 267 ± 16 gigatons of mass annually between 2000 and 2019 [5], exposing progressively nutrient-poor soils ideal for studying primary succession [6, 7]. These newly deglaciated areas are first colonized by airborne and glacially derived microorganisms, followed by cryptogams and vascular plants [8–10]. Metabarcoding has revealed that microbial communities persist throughout succession, but significant shifts in microbial composition occur along successional gradients, particularly between areas near the glacier front and those at later stages of development [9, 11]. Microbial turnover along the chronosequence has been linked to changes in ecosystem multifunctionality [12], yet the connection between microbial diversity and functional processes is still poorly understood in these low-productivity systems [13–16].

Abiotic factors are crucial in shaping microbial diversity and activity in polar terrestrial ecosystems [3, 17], but biotic interactions such as inter- and intraspecific relationships and viral/protist predation also play an important role [18, 19]. Understanding how these biotic factors interact remains a key challenge in ecology [20]. For instance, cryptogamic covers in tundra soils affect microbial composition through biogeochemical changes [21]. Additionally, microorganisms including viruses, fungi, bacteria, archaea, and protists form intricate networks involving competition, cooperation, and other interactions [22]. Viruses in particular shape microbial communities and soil processes, and influence how these communities respond to climate warming [23, 24]. Although interest in soil viruses is growing, little is known about their host interactions or how environmental conditions affect them in polar regions [25]. In fact, viruses have been recently considered “the last frontier of Antarctic diversity” [26]. Specifically, our understanding of viruses in polar glacier forefield soils is limited to a preliminary geochip-based analysis in Tierra de Fuego. This study detected 12 viral taxa with distributions varying across the successional stage along a 34-year chronosequence, suggesting that viruses may contribute to microbial populations dynamics throughout succession [12].

Long-term chronosequence studies help explain how ice-free areas are colonized after glacier retreat, but short-term studies are also essential for capturing early-stage changes in rapidly transforming landscapes. These require analysis of both taxonomic composition and microbial metabolic functions [27]. In this study, we monitored changes in the taxonomic and functional structure of microbial communities (including viruses) and soil attributes at two sites that differed by 7-years of ice-free exposure along an established chronosequence in an Antarctic glacier forefield using a metagenomic approach, combined with metatranscriptomics and qPCR analysis. To better capture early successional dynamics and the role of abiotic factors, we focused on an area with initial cryptogamic cover, minimizing direct glacial influence while allowing for some soil and microbial community development [9].

The aim of this study was to capture biotic interactions and short-term functional microbial adjustments in response to environmental fluctuations that may drive long-term successional processes. We hypothesized that microbial communities in recently deglaciated areas encompass taxonomic plasticity and a dynamic network of biotic interactions that support their ability to respond to environmental changes without compromising their functional stability. Our results reveal that microbial communities in these recently deglaciated areas possess the functional capacity to rapidly respond to biogeochemical fluctuations with minimal taxa replacement. We also identified, for the first time, a diverse set of active lytic and temperate viruses, along with abundant genes linked to antimicrobial production, resistance, and antiphage defenses. These findings highlight the importance of biotic interactions in microbial succession and the need to include them in models of colonization under climate change.

## Materials and methods

### Study area

**Figure 1:**
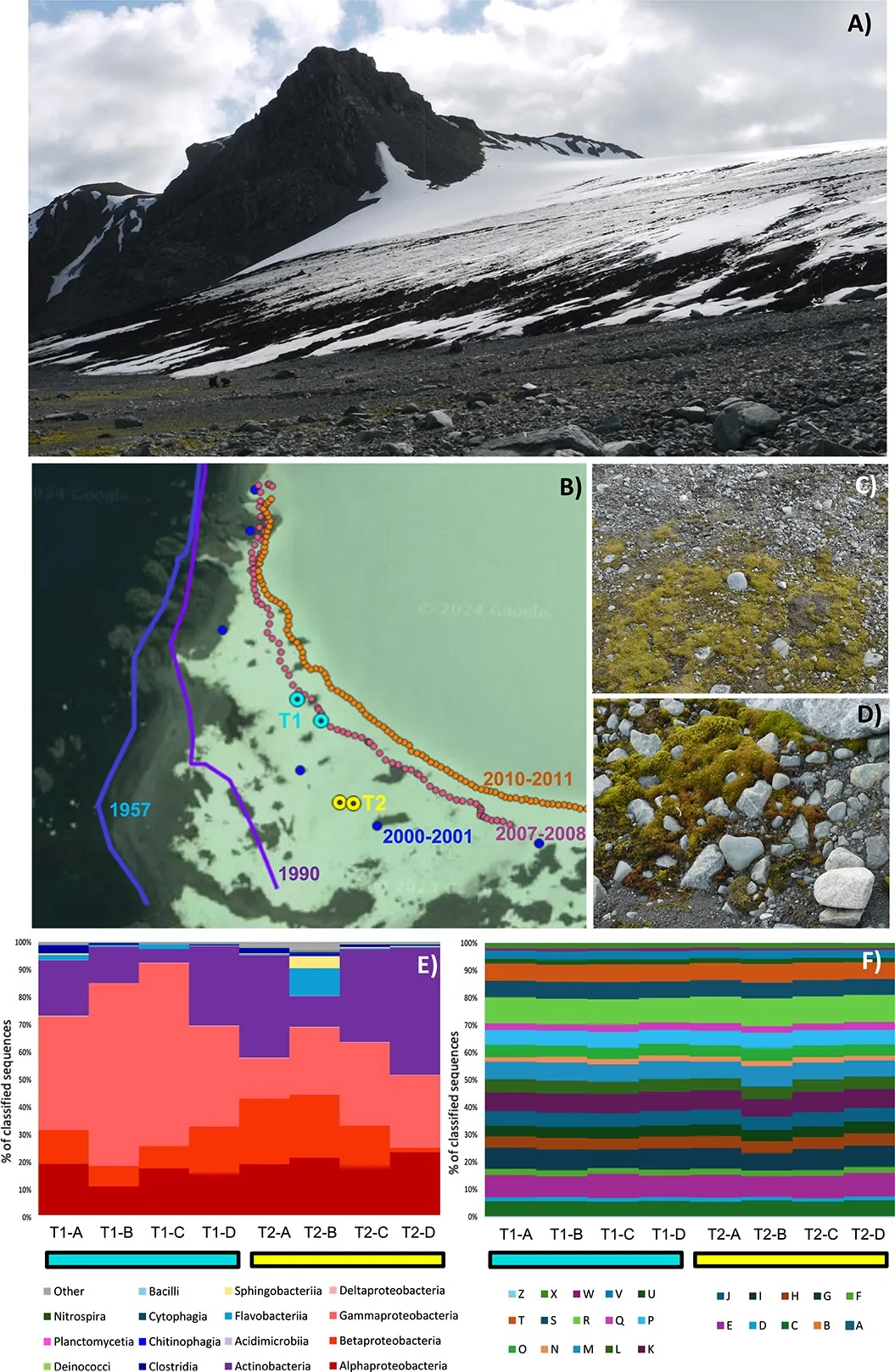
Study area and taxonomic and functional analysis. (A) Overview of the Sally rocks tongue of the Hurd glacier, located on Livingston Island, south Shetland Islands (Antarctica). (B) Changes in the terminus position of the Sally rocks tongue at the front of Hurd glacier, based on data from 1957, 1990, 2000, 2001, 2007, 2008, and 2010, 2011, as reported by Rodríguez-Cielo *et al.* (2016) and Machío *et al.* (2017).; (C, D) sampling sites showing a discontinuous moss cover, representing ~11 years (T1; C) and 18 years (T2; D) since glacier retreat. (E) Taxonomic analysis at the phylum level and functional comparison of 20 000 annotated genes per sample, recovered through metagenomics. (F) Functional annotation based on COG (clusters of orthologous groups) categories. Designations of functional categories: (A) RNA processing and modification (not used for prokaryotic COGs), (B) chromatin structure and dynamics, (C) energy production and conversion, (D) cell cycle control and mitosis, (E) amino acid metabolism and transport, (F) nucleotide metabolism and transport, (G) carbohydrate metabolism and transport, (H) coenzyme metabolism, (I) lipid metabolism, (J) translation, (K) transcription, (L) replication and repair, (M) cell wall/membrane/envelope biogenesis, (N) cell motility, (O) post-translational modification, protein turnover, chaperone functions, (P) inorganic ion transport and metabolism, (Q) secondary metabolites biosynthesis, transport and catabolism, (T) signal transduction, (U) intracellular trafficking and secretion, (Y) nuclear structure (not applicable to prokaryotic COGs), (Z) cytoskeleton (not applicable to prokaryotic COGs), (R) general functional prediction only (typically, prediction of biochemical activity), (S) function unknown.

Soil samples were collected in February 2019 from the proglacial area of the Sally Rock tongue of Hurd Glacier on Livingston Island (South Shetland Islands, Antarctica; Fig. 1A and B). The sampling area represented an early successional stage, characterized by the initial development of cryptogamic covers, with a discontinuous moss layer (Fig. 1C and D). The carbon, nitrogen, and organic matter contents (C, N, and OM respectively) reflect the oligotrophic nature of the soils (Table S1). No significant differences were found in any of these individual parameters between soils from the two exposure times (Table S1, SDS1).

The selected sites (Table S1) had been ice-free for approximately from 11 (T1) to 18 years (T2), based on documented glacier retreat measurements [28, 29] that correspond to the glacier terminus positions recorded in 2007–2008 and 2000–2001, respectively (Fig. 1B).

For each exposure time, four sampling points were established (T1 A-D; T2 A-D; Table S1) ensuring a minimum distance of 5 meters between sampling points within each exposure time. At each point, composite soil samples (0–5 cm depth) were formed from three random subsamples within 1 m × 1 m area, with moss removed beforehand. Samples were preserved in LifeGuard Soil Preservation Solution (Qiagen) and stored at 4°C. In total, eight samples were processed for metagenomics. Due to RNA extraction and cDNA sequencing issues, transcriptomic analyses were limited to T1-B and T1-D.

### Analysis of abiotic soil attributes

C and N were determined by dry combustion using an elemental analyzer (LECO TruSpec CN) at the CEBAS-CSIC. Soil OM was estimated by loss on ignition at 450°C for 4 h [30]. pH was measured using a pH-meter (Crison MicropH 2001) at a soil/water ratio of 1/10 (mass/volume).

### DNA/RNA extraction and sequencing

DNA and RNA extractions were conducted as detailed in the Supplementary Methods (SM). Library preparation (150 bp fragments) and shotgun sequencing were conducted at NOVOGENE (Cambridge, UK) on an Illumina NovaSeq PE150 platform.

### *De novo* assembly, binning, and metagenome-assembled genome analyses

Sequenced reads were quality-trimmed with *Trimmomatic* [31], and taxonomic profiling was performed using *Kaiju* [32] against the NCBI *nr* database. Reads were assembled with *SPAdes* [33], and ORFs predicted using *Prodigal* [34] (Table 1). Metabolic profiling, binning, MAG retrieval, manual curation, classification, annotation, and abundance estimation were conducted as detailed in SM.

**Table 1:** General characteristics of the metagenomes obtained in this study.

Sample	Total reads	Clean reads	base pairs (bp)	Nonpareil coverage	Nonpareil diversity (Nd)	GC %	Assembled %	Genome equivalents
T1-A (DNA)	8.04 10^7^	7.94 10^7^	1.19 10^10^	88.94	18.13	59.18	60.79	2827
T1-C (DNA)	8.38 10^7^	8.26 10^7^	1.23 10^10^	88.57	19.02	60.14	45.18	3209
T1-B (DNA)	8.06 10^7^	7.96 10^7^	1.19 10^10^	96.31	17.39	58.93	74.35	3037
T1-B (RNA)	3.71 10^7^	3.64 10^7^	5.38 10^9^	92.94	13.15	62.54		
T1-D (DNA)	8.29 10^7^	8.18 10^7^	1.22 10^10^	90.01	19.09	56.42	50.08	2819
T1-D (RNA)	5.84 10^7^	5.74 10^7^	8.59 10^9^	92.31	14.84	52.42		
T2-A (DNA)	9.31 10^7^	9.19 10^7^	1.37 10^10^	97.15	15.80	62.47	86.67	4225
T2-C (DNA)	9.79 10^7^	9.64 10^7^	1.44 10^10^	92.56	18.15	61.89	62.36	3854
T2-B (DNA)	7.90 10^7^	7.78 10^7^	1.16 10^10^	91.54	17.91	61.55	62.73	3112
T2-D (DNA)	8.72 10^7^	8.60 10^7^	1.29 10^10^	91.08	18.22	65.22	56.84	3057

### Viral sequence analyses

A stringent approach to identify viral contigs within the metagenomes was implemented using *VirSorter* [35], *VIBRANT* [36], *Kraken* v0.10.5, and *PHASTER* [37], followed by annotation and clustering into vOTUs, as described in SM.

### Metatranscriptome analysis and RNA virus recovery

RNAseq reads were quality trimmed using *Trimmomatic* [31] and assembled into contigs with *SPAdes* [33]. Cleaned reads were mapped to MAGs and viral contigs using *TopHat* 2.1.1 [38] and counted with *HTSeq* 0.6.1 [39]. Expression profiles were normalized by read count and contig length, reported as reads per kilobase pair of transcript per million mapped reads (RPKM).

Putative RNA viral genomes were identified from metatranscriptome assemblies using VirSorter2, based on the presence of the hallmark RNA-dependent RNA polymerase (RdRP) gene. A hidden Markov model (HMM) built from Pfam RdRP sequences was used for gene annotation with hmmsearch.

### Bacteria and archaea relative abundance (qPCR)

The abundances of 16S rRNA genes from Bacteria and Archaea were quantified by qPCR as a proxy for total relative abundance, as described in SM.

### Data analysis

To analyze the effects of exposure time and abiotic soil attributes (C, N and OM, and C/N) on community structure (richness and abundance of bacteria, fungi, virus, archaea, kaiju read classification, and MAGs) and different metabolic genes (Table S2), we used linear regression models (LMs) and a multimodel inference approach, as described in SM.

## Results

### Taxonomic diversity, heterogeneity, and novelty of the microbial communities in glacier forefields

Sequencing of eight soil metagenomes yielded an average of 84 million high-quality reads per sample (Table 1). Nonpareil coverage exceeded 88% in all cases, surpassing the 60% threshold for reliable assembly and gene detection [40]. Massive pairwise analysis (MASH) of raw reads showed no clear clustering by exposure time (Fig. S1A). Sequence diversity indexes (Nd) retrieved from nonpareil analysis [41] spanned from 15.80 to 19.09 (Table 1), a broad range that reflects the heterogeneity of our samples. The model including soil OM best explained the Nd values, accounting for 60.3% of the variance, with a negative effect (Table 2, SDS1).

**Table 2:** Variance partitioning of the best models (selected based on AICc values) assessing the effects of abiotic factors and exposure time on different response variables. A complete list of tested models is available in Table S3. Negative values denote a negative relationship.

			**Exposure time**	**C** **content**	**C/N** **ratio**	**OM** **content**	**N** **content**	**pH**
**Whole metagenome analyses**		Nonpareil diversity (Nd)				**−60.3**		
		Genome equivalents	**38.8**			**24.1**		
		Abundance taxa all reads	**43.3**					
		Bact 16S rRNA gene richness	**−61.3**	**−28.3**				
		Bact 16S rRNA gene abundance			**18.4**			
		Fungal read richness		**−51.3**				**7.6**
		MAG richness					**−50.4**	**20.4**
		MAG abundance	**30.4**			**63.5**		
		Abundance COGs reads	**24**		**24.6**	**20.2**		
		Lytic viruses richness	**−9.4**			**−14.2**		**62.1**
		Lytic viruses abundance	**−28.8**					
		Prophage richness	**−32.8**				**−58.2**	
		Prophage abundance	**−34.6**	**27.7**				
**Specific** **genes** **analysis**	Carbon cycle	Hidrogenase			**27.4**			
		Rubisco			**26.9**			
		PRK				**−88**		**15.1**
		Citrate lyase				**79.05**		
		Piruvate lyase				**−31.6**		
		Fumarate/succinate reductase	**31.6**	**−14**				
		Carbon monoxide dehydrogenase				**−86**		
		Lactate dehydrogenase	**43.9**					
		cytochrome c oxidase subunit I				**78**		
		cytochrome c oxidase subunit II				**82.9**		
		Metanooxigenase (Mmbo)			**17.6**			
	Phototrophy	Bacterial rhodopsin				**−53.8**		
		Heliorhodopsin				**−47.9**		**10.6**
		pufM				**−31.7**		**24.5**
		phycobilisomes				**−42.1**		
	Nitrogen cycle	nifH					**11.8**	**−22.1**
		Nitrate_red_gam	**−5.6**	**−92.8**				
		Nitrite reductase	**−23.6**		**−21.4**			
		NosL				**18.2**		
	Sulphur cycle	sulfate adeylyltransferase	**68.5**				**25.4**	
		SOXY		**43.3**				
		Sulfite reductase		**28.9**				

The number of genome equivalents, a proxy for cell abundance [42], was estimated for each sample (Table 1). Its variance was best explained by exposure time (38.8%) and soil OM (24.1%).

Taxonomic analysis (Fig. 1E, SDS2) showed that the communities were mainly composed of Bacteria (98.8%–99.94% of the reads), followed by Eukarya (0.02%–1.12%), while Archaea (all belonging to the Euryarchaea) accounted for 0.02%–0.4% of the reads. Differential abundance analysis confirmed bacterial dominance, with qPCR data from 10 additional samples (Table S4), revealing a 2- to 3-order magnitude difference in 16S rRNA gene abundance between domains. Within Bacteria, Pseudomonadota was the most abundant phylum, followed by Actinomycetota, Bacteroidota, Bacillota, and Acidobacteriota, while Cyanobacteriota were scarce (<0.04% reads). Dominant genera included *Pseudomonas*, *Lysobacter*, *Devosia*, *Cellulomonas*, and *Brevundimonas*. The community structure was heterogeneous, but exposure time influenced composition, explaining 43.3% of total read abundance variance and 61.3% of bacterial 16S rRNA gene richness variance (Table 2, SDS1). Indicator species analysis identified the following low-abundance taxa significantly associated with the T1 exposure period (*P* <0.05): the bacterial phyla Fusobacteriota, Fermentibacterota, Margulisbacteria, and Caldisericota, the protist phylum Evosea and the archaeal phyla Iainarchaeota, and Aenigmarchaeota (Table S5).

Fungal reads ranged from 0.01% to 0.51%, corresponding mainly to Ascomycota, particularly the class Leotiomycetes (Fig. S1B), with minor representation of Basidiomycetes. C explained 51.3% of the variance in fungal richness (Table 2, SDS1).

Assembly of reads into contigs resulted in 546 534 contigs larger than 1 kb, which constituted a large proportion of the assembled reads (45–85%, Table 1), indicating low intraspecific diversity. From the 685 Mbp assembled data, we reconstructed a dereplicated set of 52 high-quality and 19 medium-quality MAGs, all manually curated, which recruited between 38% and 70% of the total reads. To minimize taxonomic missassignments and incomplete pathway reconstructions [43–45], we used only high-quality MAGs (SDS3). Taxonomic classification based on best amino acid identity (AAI) against available reference genomes [42] revealed that the MAGs belonged to Pseudomonadota (26 MAGs), Actinomycetota (15), Bacteroidota (8), Bacillota (2), and Patescibacteriota (1), reflecting the community structure patterns observed in the read-based analysis (Fig. 1E).

The 52 high-quality MAGs showed 80%–100% completeness (average 93%) and low contamination (0%–4.2%, average 0.99%) (SDS3). Genome sizes ranged from 1.7 Mb (Saccharimonadaceae) to 7.2 Mb (*Cypionkella*), with GC content between 31% and 70%. Only one MAG (MAG48) had >95% ANI to a known species, *Janthinobacterium svalbardensis*, previously isolated from a Svalbard glacier [46]. The rest belonged to uncultured genera, indicating high microbial novelty.

Nearly half of the MAGs (21/52) appeared in only one sample, underscoring sample heterogeneity. Others, like *Lysobacter*, Burkholderiaceae, and *Devosia*, were more widespread. MAG abundance was best explained by OM (63.5%) and exposure time (30.4%), though neither significantly affected MAG richness (Table 2 and SDS2).

**Figure 2:**
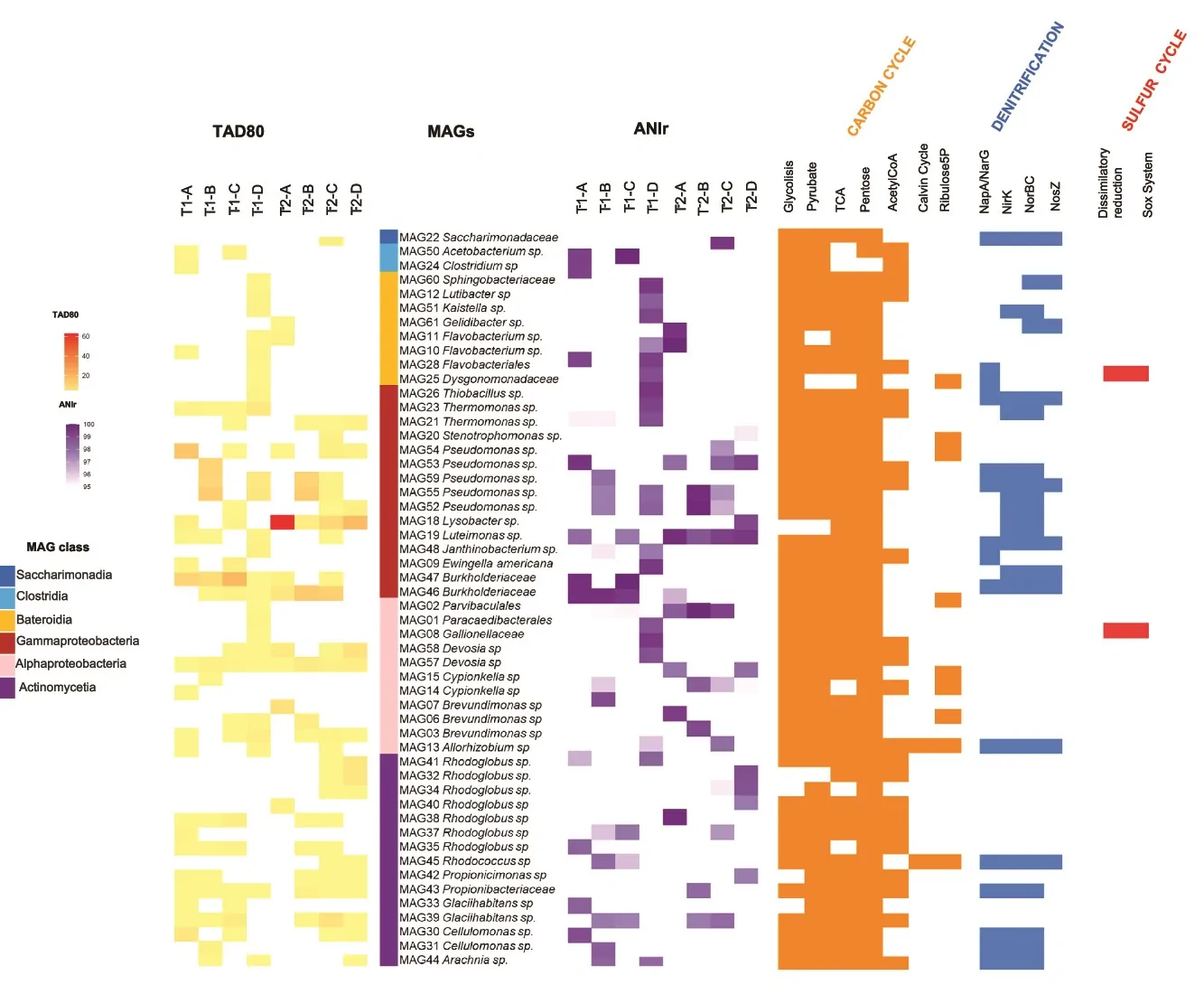
Heatmaps of MAG relative abundance, ANIr values, and metabolic pathways in Antarctic soil metagenomes. The left panel shows the relative abundance of MAGs in each sample, calculated as the truncated average sequencing depth (TAD80) normalized by genome equivalents. The central panel presents the average nucleotide identity for reads (ANIr) from each metagenome mapped to each MAG. The right panel displays metabolic pathways associated with carbon, nitrogen, and sulfur cycling. The column between the TAD80 and ANIr panels indicates the phylogenetic classification of MAGs color-coded according to the MAG class legend.

To assess the diversity or clonality within MAG-defined populations, we calculated average nucleotide identity of mapped reads (ANIr) [47]. MAGs found only in one sample had high ANIr values above 97% (Fig. 2, SDS3), suggesting recent colonization. MAGs from abundant genera like *Pseudomonas* and *Lysobacter* also showed high ANIr (>95%), indicating homogenous populations. In contrast, *Cellulomonas* and *Devosia* MAGs had ANIr below 95%, suggesting the presence of genetically distinct co-occurring populations.

We estimated maximum generation time (Tg) for each MAG using codon usage analysis [48], which ranged from 0.17 to 10.14 h (average 2.47 h; SDS3). MAGs found in single samples showed the greatest Tg variability. The remaining MAGs had an average Tg of 2.02 ± 0.77 h.

Metatranscriptomic analyses of samples T1-B and T1-D (SDS3) revealed a strong correlation between MAG abundance (TAD80/genome equivalents) and expression (Pearson r = 0.8 and 0.4, respectively), indicating that the most active MAGs were also the most abundant.

### Functional profiles of the microbial communities

Functional profiles based on COG classification of unassembled reads were more homogeneous than taxonomic profiles (Fig. 1F). Exposure time explained 24% of COG abundance variance, with C/N (24.6%) and OM (20.2%) also contributing, highlighting the interaction with soil attributes (Table 2, SDS1).

**Figure 3:**
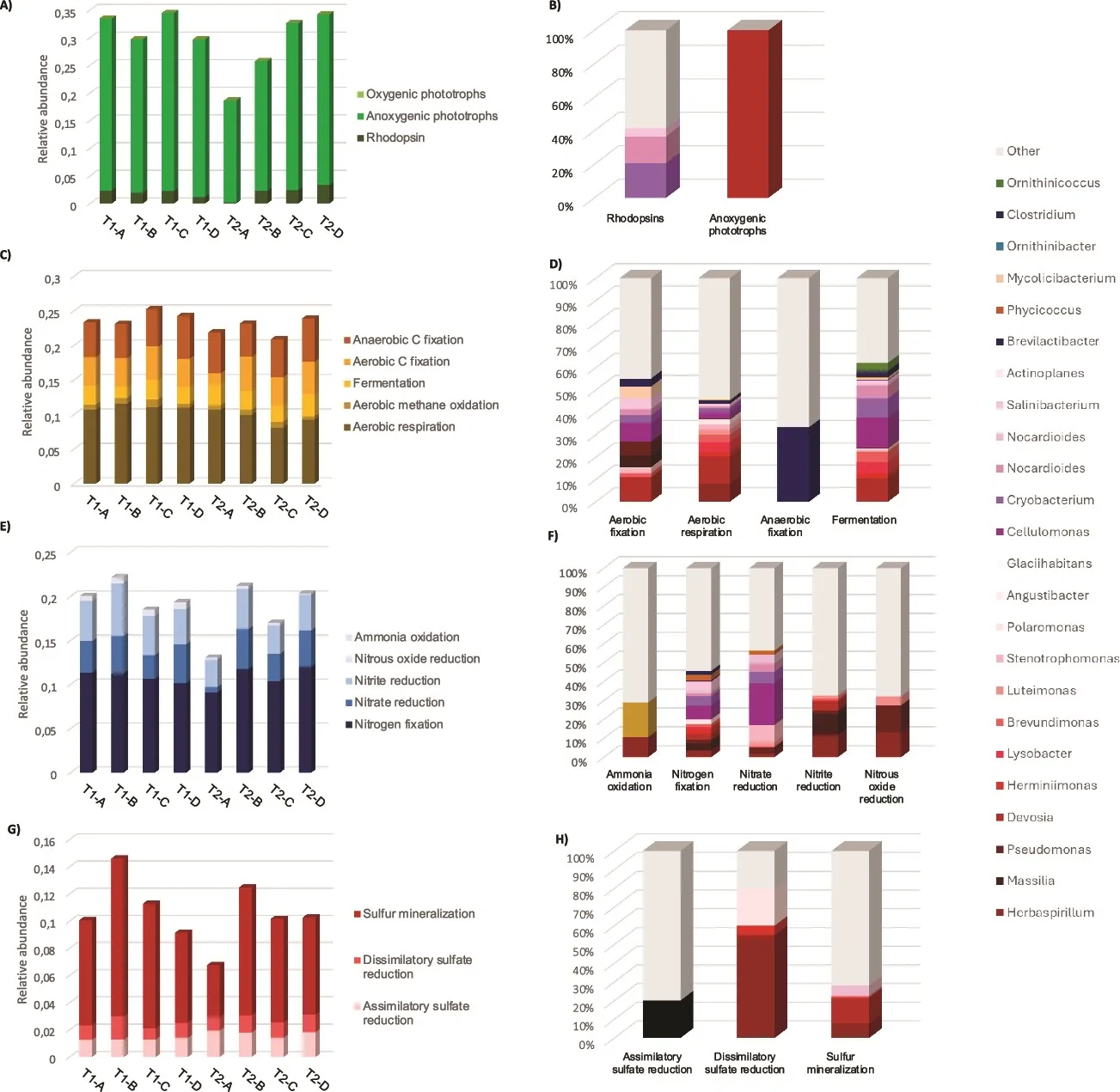
Main metabolic pathways and associated taxa in Antarctic soils. The left panel illustrates key prokaryotic metabolic processes, including phototrophy, and the carbon, nitrogen, and sulfur cycles. The right panel displays the major taxa involved in each process. The genetic potential for each conversion step was estimated using a combination of normalized marker genes (SDS5). (A) Prokaryotic phototrophy and (B) its major taxa. (C) Carbon cycle and (D) its major taxa. (E) Nitrogen cycle and (F) its major taxa. (G) Sulfur cycle and (H) its major taxa.

To assess metabolic strategies, we analyzed the distribution and taxonomy of 24 conserved biogeochemical marker genes, identified from contigs (Fig. 3, SDS4), based on prior studies in Antarctic rock and soil environments [49].

#### Phototrophy

Markers for aerobic anoxygenic phototrophs were most abundant, while rhodopsin genes were less common and oxygenic phototrophy markers were nearly absent (Fig. 3A). Anoxygenic photosynthesis was mainly linked to Alphaproteobacteria, with over half of *pufM* genes assigned to *Devosia* (Fig. 3B). Gene abundances for *pufM* and rhodopsins were inversely correlated with OM (Table 2, SDS1).

#### Carbon cycle

Genes involved in aerobic carbon fixation were detected, with RuBisCO primarily found in Pseudomonadota and prkB mainly in Actinomycetota (Fig. 3C and D). Fermentation genes such as *ldh* were less abundant than those for aerobic respiration (Fig. 3C). Aerobic respiration (Fig. 3D) was mainly linked to Pseudomonadota (*Devosia*, *Pseudomonas*, and *Lysobacter*), while fermentation genes were associated with Actinomycetota (*Cellulomonas* and *Cryobacterium*)*.*

Unexpectedly high numbers (i.e. over 100) of genes for xenobiotic degradation and secondary metabolism were found in several MAGs, particularly those related to *Pseudomonas* (SDS5 “Selected categories”). For context, the known degrader *Stutzerimonas chloritidi*s *mutans* carries 116 such genes, while *Pseudomonas* and *Rhodococcus* MAGs had 151 and 124, respectively.

#### Nitrogen cycle

Metagenomes revealed genes for N_2_ fixation, nitrification, and denitrification (Fig. 3E), with *nifH* being most abundant and mainly linked to Actinobacteria (*Cellulomonas*, *Cryobacterium*) (Fig. 3F). Only one MAG (Burkholderiaceae) encoded the full nitrogen fixation pathway.

*nirK* genes were the most abundant denitrification markers, mainly in *Cellulomonas*. *nosL* genes were less common and found mostly in *Pseudomonas* and *Devosia* (Fig. 3F). Seven MAGs from diverse taxa (e.g. *Rhodococcus*, *Allorhizobium*, and *Janthinobacterium*) encoded complete denitrification pathways. Sixteen additional MAGs had partial pathways, with 10 lacking *nosZ*, suggesting incomplete denitrification capacity. Variation in nitrogen-cycling genes was partly explained by soil attributes related to carbon cycling (Table 2, SDS1).

#### Sulfur metabolism

The sulfur mineralization marker gene (*sir*) was the most abundant, mainly associated with *Devosia* (13%) and *Pseudomonas* (7%) (Fig. 3G and H). A complete sulfur oxidation system (*Sox*), was detected in two Betaproteobacteria MAGs (*Thiobacillus* sp. and a Gallionellaceae member). C explained 43.3% of the variance in *Sox* gene abundance and 28.9% for *sir* genes, both showing positive effects (Table 2, SDS1).

#### Microbial resistance and secondary metabolite synthesis genes

Assembled metagenomes contained an average of 272 000 ORFs, with 0.75%–0.86% annotated as antibiotic resistance genes (ARGs), spanning 26 drug classes. The most common ARGs were for multidrug resistance (32.8%), macrolides (14%), tetracycline (8.5%), peptides (8%), and glycopeptides (6.1%). Efflux pumps (67.4%) and antibiotic target alteration (12.3%) were the main resistance mechanisms. These results are shown in detail in SDS4, “Antibiotic resistance genes” tab. “Drug resistance: antimicrobial” pathways were found in all MAGs, with *Lysobacter*, *Stenotrophomonas*, *Janthinobacterium*, *Pseudomonas*, and *Ewingella* (SDS5) carrying the highest numbers (30–51 genes), similar to multiresistant *Pseudomonas* strains (29–53 genes) based on BLAST-Koala analysis.

Several MAGs also showed considerable potential for antimicrobial compound synthesis, as shown in SDS5 (“Selected categories”). For instance, *Pseudomonas aeruginosa*, notorious for this ability [50], harbored 41 genes classified under “Metabolism of terpenoids and polyketides” and 61 in the category “Biosynthesis of other secondary metabolites”, while the number of such genes in some of our MAGs were as high as 35 and 53, respectively.

### Diversity, abundance, and activity of virus

Using a stringent approach, 106 DNA viral contigs were recovered from the eight soil metagenomes and grouped into 91 viral operational taxonomic units (vOTUs), comprising 63 lytic (i.e. virulent) and 28 prophages (SDS6). Only 11 vOTUs contained more than one contig, each originating from different samples. Like MAGs, almost half (49%) of vOTUs appeared in only one sample, and none in more than five, reflecting strong spatial and temporal heterogeneity. 14 prophages were identified within MAGs, some containing up to five. Individual MAGs and their corresponding prophage abundances were highly correlated (r = 0.95). Lytic viruses were less abundant than prophages, based on metagenomic data. Exposure time explained up to 28.8% of the variance in lytic virus metrics and over 34% for prophages (Table 2, SDS1).

**Figure 4:**
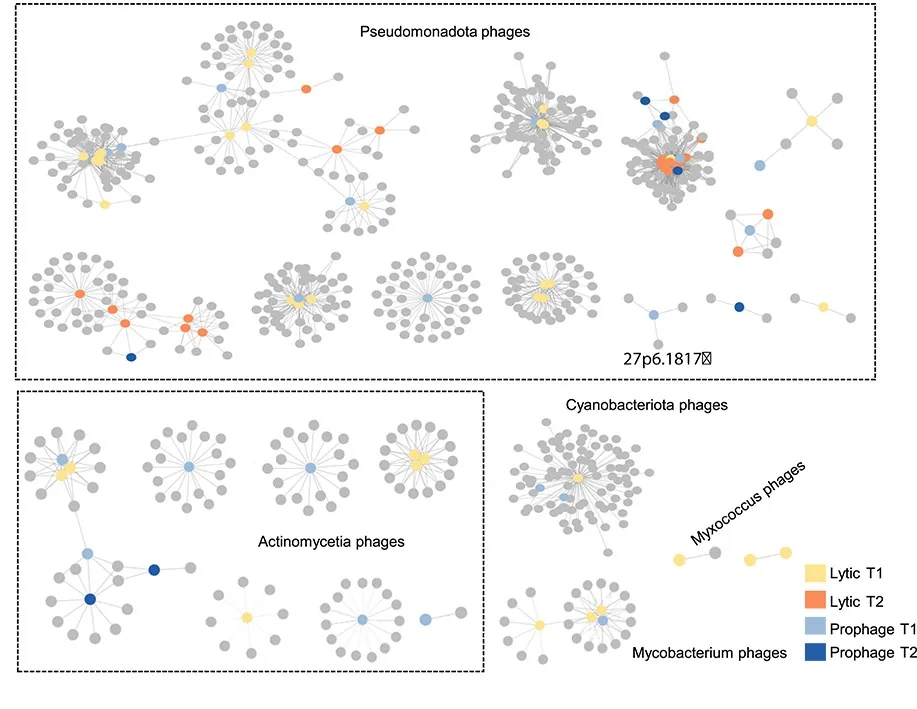
vConTACT clustering of phages and prophages and related prokaryotic viral RefSeq genomes (version 201). Color nodes represent the predicted viral sequences and grey ones the RefSeq virus. Edges represent the vConTACT-generated similarity score between each pair of viruses (only similarity scores of ≥1 are included in the network). Highly similar viruses are positioned close together. Only reference viruses that are connected to ≥1 predicted phage are included in the network.

VIRIDIC analysis [51] revealed high genomic heterogeneity among soil glacier viruses, with only one vOTU pair exceeding the 70% similarity threshold for genus-level classification. Phylogenomic trees based on protein sequences supported this, showing no clustering among our vOTUs or with known viruses (Figs S2–S4). Similarly, vCONTACT analysis showed limited connectivity: only 18 of 28 prophages and 45 of 63 lytic vOTUs clustered with other viruses (Fig. 4), underscoring their novelty and diversity. Most clusters included viruses recovered at a given time point, either prophages or lytic, in good agreement with the influence of exposure time on virus metrics mentioned above.

As with MAGs, ANIr values were calculated by mapping metagenomic reads against lytic vOTUs (SDS6) as a proxy of population heterogeneity. Only 12 of 63 vOTUs (SDS6), showed ANIr below 99% indicating low intra-population variation. Most of the 52 vOTUs found in multiple samples remained highly homogeneous, with ANIr above the 99.2–99.8% threshold for defining viral genomovars [52]. A positive correlation between virus abundance and ANIr suggested high clonality among actively replicating viruses across samples, even when detected at both exposure times.

Hosts were assigned to 25 (out of 63) and 22 (out of 28) lytic and prophage vOTUs, respectively. For prophages within MAGs (SDS6), our assignments matched MAG taxonomy, supporting the approach.

The viral assemblage in this glacier forefield showed high genetic diversity (Table 3, SDS6), with 11.9% of the genes with known functions in the lytic genomes and 56.6% in the prophages. Many genes encoded typical viral traits (Table 3), but a notable fraction represented cellular functions, including accessory metabolic genes (AMGs) that may boost host fitness [53]. Using a strict criterium, we excluded AMGs related to nucleic acid and carbohydrate metabolism due to likely viral roles. AMGs were more frequent in prophages (10.9% of the total genes in prophage genomes) than in lytic vOTUs (1.4%). Many AMGs were embedded in viral regions and showed high similarity to bacterial homologues, suggesting recent transfers. Additionally, toxin-antitoxin systems were more common in prophages than in lytic vOTUs, consistent with their role in stabilizing prophage integration within host genomes [54].

**Table 3:** dsDNA viruses recovered *in silico* from the analyzed metagenomes.

	**Prophages** N°	%	**Lytic viruses** N°	%
**Number of bona fide viruses (all sites)**	**31**		**75**	
**Number of vOTUs (all sites)**	**28**		**63**	
Size range (all sites) Kpb	13 569–135 821		10 058–321 419	
Associated to host	16		25	
**Detected protein coding genes**				
**Automatic classification**	**1681**		**4264**	
Potential AMG (including CHP)	815	48.5	1955	45.8
VOG	866	51.5	2309	54.2
**Manual inspection**				
Annotated^*^	952	56.6	509	11.9
DUFs and detected domains	144	8.6	110	2.6
Phage related genes^**^	360	21.4	57	1.3
Nucleic acid metabolism and regulation	188	11.2	236	5.5
GTAs	1	0.1	5	0.1
Lytic enzymes, CAZymes	73	4.3	39	0.9
Cellular function/”bona fide” AMG	183	10.9	61	1.4
Transport related	23	1.4	1	0.02
Toxin/antitoxin //ParB	5	0.3	1	0.02

^*^With function ^**^Not in other categories.

Metatranscriptome mapping in samples T1-C and T1-D showed all vOTUs were active (SDS6). A distinct differential pattern was observed between lytic viruses and prophages when comparing their ratio of (normalized) reads recruited from metatranscriptomes versus metagenomes, since lytic viruses consistently displayed higher ratios (3.059 ± 5.656 for lytic versus and 0.006 ± 0.006 for prophages). This suggests lytic viruses were actively replicating, while prophages remained mostly dormant within host genomes despite higher genomic presence. To check the transcription levels of transport genes encoded in prophages, we compared normalized transcription of transporter genes to their respective prophage genomes in the two metatranscriptomes. In 12 of 13 cases (SDS6, tab: transporter expression), the ratio exceeded one (up to 53) indicating selective gene expression.

RNA viruses were explored in the two metatranscriptomes by assembling contigs containing the RNA-dependent RNA polymerase (*RdRp*) gene. This approach identified 116 contigs, clustered into 106 vOTUs (SDS7), with only nine shared between samples. Tentative host assignments suggested most vOTUs infect bacteria, though some were linked to eukaryotes such as plants, invertebrates, and fungi. Most belonged to the +ssRNA group. Like dsDNA viruses, the RNA viral community was highly diverse and only distantly related to known viruses (Fig. S4).

### Defense systems: CRISPR analysis unveiled virus-host dynamics along glacier retreat

Except for MAG34, which lacked identifiable antiphage defense genes, MAGs showed up to two orders of magnitude variation in the abundance of defense systems (SDS5, tab “selected categories”). The highest densities were observed in MAG24 (*Clostridium*), MAG25 (Dysgonomonadaceae), and MAG26 (*Thiobacillus*) with values of 1.11 × 10^−2^, 1.25 × 10^−2^, and 1.84 × 10^−2^ per kb, respectively. For context, defensome studies report gene densities ranging from none (in intracellular bacteria and obligatory endosymbionts) to over 8 × 10^−3^ in the human gut and around 1.5 × 10^−2^ in soil and marine environments [55]. Restriction-modification systems were the most common, representing ~40% of the identified defense genes.

Among the defense systems identified, the presence of CRISPR-Cas (Clustered Regularly Interspaced Short Palindromic Repeats and associated proteins) in MAGs was modest but given their unique role in elucidating virus-host dynamics in natural systems, we explored them in depth in our metagenomes. CRISPR-Cas provides adaptive immunity in prokaryotes by using viral DNA fragments (spacers) stored in CRISPR arrays to recognize and defend against future infections, offering insights into virus-host interactions and ecological dynamics [56, 57].

A total of 91 CRISPR arrays containing 1180 spacers were identified across the eight metagenomes, averaging 13 spacers per array, which is within the optimal number of spacers, previously suggested to range between 10 and 100 spacers within bacterial genomes (Ref 58 in 63). Most arrays (over 90%) had 1–50 spacers (Fig. S5). High viral mutation rates can render older spacers ineffective, favoring shorter arrays, while greater viral diversity reduces the efficiency of CRISPR-Cas but does not necessarily result in longer arrays. Thus, it seems that fighting against multiple viral species with low mutation rates favors arrays with more spacers that allow the cell to increase the probability of survival [58]. This may apply here, as high ANIr values suggest clonal viral populations co-occur with high spacer counts. To assess CRISPR-Cas activity, spacers were mapped against assembled viral contigs, but no matches were found, implying active viruses evade CRISPR targeting or that the system works and keeps targeted viruses from replicating. This aligns with the expectation that CRISPR systems would inhibit the activity of targeted viruses. Only one spacer matched the *RefSeq* viral database, highlighting the novelty of the virome.

**Figure 5:**
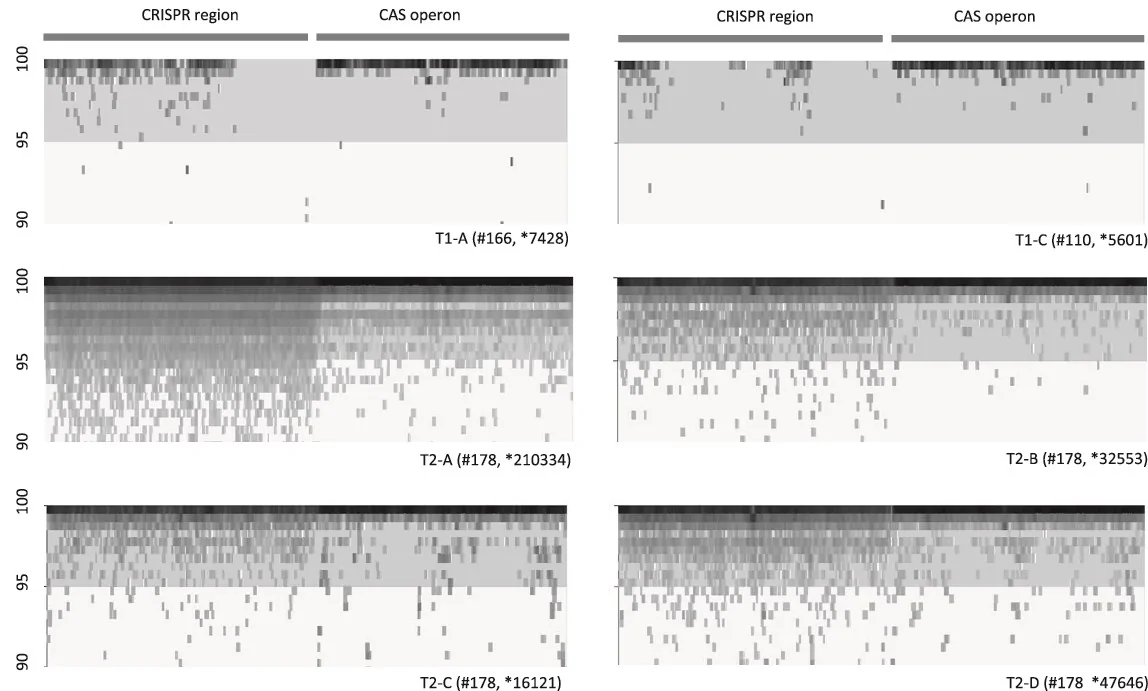
Spacers in *Lysobacter* MAG18. Fragment recruitment plot of the *Lysobacter* contig containing CRISPR-Cas elements at different locations. Short reads from selected sample metagenomes (T1–A, T1-C, T2-A, T2-B, T2-C, and T2-D) were aligned against the *Lysobacter* contig in a BLAST search, and the recruitment plot illustrates where individual metagenomic reads matched it (x axis), along with the percentage identity of the match (y axis). Numbers preceded by # indicate the total number of spacers detected, whereas numbers preceded by ^*^ indicate the number of recruited reads.

A complete and active CRISPR-Cas system was found in MAG18, corresponding to *Lysobacter* sp. This system contained unique spacers near the 5′ end of the array at T2, not present at T1 (Fig. 5), indicating acquisition of new spacers over exposure time. The acquisition of spacers was accompanied by a sharp increase in MAG18 abundance at T2 (Fig. 5), peaking at 628 TAD80 (Fig. 2, SDS5) in sample T2-A which was the highest value for any MAG in any of the eight metagenomes.

## Discussion

This study offers a comprehensive view of the taxonomic and functional composition of microbial communities in forefield maritime Antarctic soils (11–18 years free of ice), before significant colonization by higher plants. We observed a highly heterogeneous and responsive community, composed of both cosmopolitan and potential transient taxa with varying activity and generation times.

Microbial communities established in the analyzed soils recently exposed by glacier retreat exhibited low diversity, as indicated by Nd calculated from unassembled reads [41]. These values are consistent with those reported for maritime Antarctic soils and are lower than those typically observed in nonpolar regions [59, 60]. Sample heterogeneity was also evident and may reflect underlying biogeochemical differences. As variations in OM, C, and N cannot be solely attributed to exposure time or soil development, this variability may result from the uneven deposition of organic inputs, such as sporadic contributions from marine mammals or seabirds [61]. Indeed, in our study, organic matter was negatively correlated with microbial diversity, possibly due to the presence of toxic compounds in guano that inhibit the growth of certain microbial species [62].

The dominance of Pseudomonadota and the high abundance of Actinomycetota, have also been reported in other polar glacier forefields [63, 64]. However, the low abundance of Cyanobacteriota is not a consistent trait across these systems [63, 65, 66] which suggests site-specific distributions for this phylum.

The observed dominance of Leotiomycetes is consistent with previous studies, which identified this class as the most abundant and species-rich group of fungi in Antarctic soils [67]. Leotiomycetes have been associated with plant root system development in polar regions [68], which may explain the observed relationship between their richness variability and C. The high proportion of unidentified sequences, indicating the presence of numerous novel taxa, is consistent with findings from other Antarctic soil studies [14, 63].

The estimated patterns of maximum generation time suggest a relatively fast growth rate for environmental microorganisms and indicate a potential for rapid response to changes associated to glacier retreat [69]. These rates contrast with those of slower-growing MAGs from the more extreme, nutrient-poor environments of the Antarctic Dry Valleys [70]. In turn, metatranscriptomic analyses confirmed active community members during the environmental fluctuating Antarctic summer [71], when these samples were collected. This was especially true for potential recent colonizers with high ANIr values found in only one sample. For instance, MAGs 01, 25, 51, and 60 (affiliated with Paracaedibacterales, Dysgonomonadaceae, *Kaistella*, and Sphingobacteriaceae) showed >99% ANIr and expression across ≥70% of their genomes (Table T1-B and T1-D, SDS3). These microorganisms may respond quickly to environmental fluctuations but enter dormancy under harsher winter conditions, a common survival strategy in glacier environments [72]. However, some may also be transient rather than persistent members, as noted in other glacier forefields [9, 11, 73]. In fact, the presence of indicator taxa in T1 soils (Table S5), which are characterized by shorter exposure times, suggests a replacement of taxa between the two successional states analyzed. T1-associated taxa may include microorganisms originating from subglacial aquatic environments [74], which are likely transported by meltwater to areas near the glacial front but become less detectable in more exposed soils due to challenges in establishment [75]. Microbial biomass, estimated by the number of genome equivalents, appeared to increase with longer exposure times and greater OM, as in other glacier forefields [76].

Functional profiles have been shown more homogeneous than taxonomic profiles. This functional homogeneity supports the idea that functionally cohesive microbial groups are more stable and linked to metabolic activity than individual taxa [77]. The detection of similar metabolic pathways across taxonomically distinct microbial taxa suggests functional redundancy [28, 78, 79], a pattern rarely explored using metagenomics in polar soils [80].

Sunlight may be a key environmental driver of these microbial communities, as suggested by the predominance of genes related to anoxygenic photoheterotrophy, particularly those associated with the genus *Devosia*. Though less abundant, rhodopsin-dependent photoheterotrophy, primarily linked to certain Actinobacteria, aligned with earlier findings of photoheterotrophic activity in nutrient-poor environments [21, 81]. In contrast, genes associated with oxygenic phototrophs were scarce, consistent with the low abundance of cyanobacteria observed.

In these energy-limited glacial soils, microorganisms may rely on both anoxygenic and retinal-based phototrophy to harvest solar energy during the summer months, sustaining a predominantly heterotrophic metabolism [82]. In fact, genes related to heterotrophic pathways are more prevalent than those involved in CO₂ fixation in these communities. The seasonal input of glacial meltwater may further promote the establishment of heterotrophic microorganisms by supplying allochthonous carbon sources during the summer months [83]. The notable capacity for xenobiotic degradation found may also support the breakdown of old, recalcitrant organic matter from exposed previously trapped glacial deposits [84] providing an additional carbon source for heterotrophic microorganisms. MAGs with xenobiotic degradation genes also showed many transport, motility, and environmental response genes, indicating high adaptability to oligotrophic and dynamic glacier forefield environments [85].

Regarding the nitrogen cycle, incomplete denitrification pathways, particularly those lacking nitrous oxide reductase genes, appear more prevalent than complete pathways in both metagenomes and recovered MAGs. This suggests that microbial communities in these soils may contribute to the release of nitrous oxide, a potent greenhouse gas. As shown in various genome and metagenome analyses [86, 87], only a subset of denitrifying microorganisms encoded the full enzymatic machinery required for complete denitrification. Truncated pathways are also frequent in nitrogen-limited polar tundra soils with fluctuating nitrate levels [88], and have been observed in Antarctic soils impacted by marine animals, where incomplete denitrifiers are more prevalent [89]. These communities also harbor heterotrophic microorganisms capable of degrading organic sulfur, with penguin guano likely contributing to soil sulfur and other bioelement inputs through deposition [49, 61].

ARGs were highly prevalent in our samples, with multidrug resistance mediated by efflux pumps being the dominant resistance mechanism, consistent with findings from other Antarctic soils [71, 90–92]. The low human impact in these areas suggests that ARGs likely originate from natural adaptations to environmental stressors [12]. Co-evolution in extreme habitats may favor ARGs that provide broader stress tolerance in addition to antibiotic resistance [93], suggesting strong interspecies competition. However, in the absence of direct evidence, alternative roles for antibiotics, such as acting as signaling molecules [94] or serving as nutrient sources [95], cannot be excluded. These findings underscore the importance of chemical interactions in shaping microbial communities in glacier forefields.

The viral community exhibited a high degree of novelty. None of the vOTUs identified in this study matched known sequences from other Antarctic environments, including the over 75 000 vOTUs previously recovered from Antarctic rocks, the largest existing collection to date [96]. This supports prior findings of high viral heterogeneity in Antarctica [97], but contrasts with Arctic studies where identical viral genomes were found across sites [98]. The relatively low vOTU count in our study, compared to other polar environments studies, likely reflects the strict criteria we used to define viral contigs.

*Pseudomonas*, the most common genus in metagenomes, was also the most frequent host for recognized virus, suggesting virus-host interactions may influence its diversity and dynamics. In contrast, no viruses were linked to *Lysobacter*, despite its high abundance, a point discussed below. The high proportion of prophages among detected vOTUs suggests that part of the viral community may follow a Piggyback-the-Winner strategy, aligning with a microbial community marked by bursts of growth and productivity [99]. Conversely, viruses linked to hosts like *Microbacterium*, *Roseobacter*, and *Streptomyces* appeared strictly lytic, indicating host-specific strategies.

Viral contigs showed a notable prevalence of genes for transport functions, including both modules of ABC (ATP-binding cassette) transporters which are ATP-dependent membrane systems present in all cellular life [100]. While membrane transport genes have been reported in the ocean virome [101], their abundance was lower than in our dataset. Despite being known in viral genomes, ABC transporters remain uncommon [102; our database search]. The presence of prophage-encoded transport genes may represent an adaptation to the oligotrophic conditions of recently deglaciated soils (C: 0.05%–0.32%). These genes could offer a competitive advantage to host bacteria. Similar findings in Arctic viral metagenomes suggest ABC transporters may aid nutrient acquisition [103], as suggested by the metatranscriptomic analyses. These results support the idea that viruses may enhance host survival in various polar oligotrophic environments, as proposed for the Antarctic Dry Valleys [104].

Antiphage defense genes were detected in the majority of MAGs obtained in this study. The prevalence of defense genes classified as restriction–modification systems is consistent with findings in soil communities from previous studies [104]. In contrast, BREX (BacteRiophage EXclusion) and DISARM (Defence Island System Associated with Restriction-Modification), were the dominant antiphage immunity mechanisms identified in Antarctic desert hypoliths.

Among the defense systems identified, the presence of CRISPR-Cas in the MAGs may be particularly relevant due to its potential to shape microbial interactions within these communities. In a recent study of CRISPR-Cas systems in the pristine poly-extreme McMurdo Dry Valley Antarctic soils, the largest number of spacers found in the 18 MAGs retrieved was 50, which was interpreted as spatially constrained phage-host interactions [70]. This contrasts sharply with the exceptionally high number (178) of spacers found in MAG18 that was recovered from soils after 18 years of glacier retreat. The sharp increase in MAG18 abundance at T2, along with the absence of detectable protospacers in the metagenomes and the lack of viruses assigned to *Lysobacter*, suggests a highly effective CRISPR-Cas system. In contrast, virus-host interactions within the frequently retrieved *Pseudomonas* assemblage likely involved a high diversity of active viruses, including both lytic and prophage forms. These findings indicate that the overall heterogeneity observed in the analyzed glacier forefields extends to the interaction strategies between virus and their hosts, even within the same phylogenetic groups.

## Conclusions

These communities, dominated by Pseudomonadota and characterized by low abundance of photosynthetic microorganisms, harbored both cosmopolitan and potentially transient taxa with varying levels of activity and generation times. Successional changes were evident, with some potentially transient low-abundance taxa serving as indicators of the youngest soils. The presence of active, potentially transient microorganisms suggests that these taxa are crucial in adapting to environmental fluctuations. However, heterogeneity in taxonomic community structure, in both virus and cell assemblages appears to be primarily driven by fine-scale biogeochemical factors. Nonetheless, we cannot rule out site-specific contingencies influencing microbial community composition that are not accounted for in the space-for-time substitution assumption underlying chronosequence studies. This contrasts with the more homogeneous functional diversity, which likely reflects functional redundancy supporting a broad range of metabolic pathways, strongly influenced by soil attributes.

An active, highly novel, and heterogeneous assemblage of lytic and temperate viruses was detected in all the samples, with hosts assignable in half of the cases. Notably, prophages may play a role of in regulating host fitness through the expression of membrane transporters encoded in the viral genome. Coupled with the high abundance of genes involved in antimicrobial compounds synthesis and resistance and numerous anti phage defense systems, these findings underscore the role of biotic factors in driving successional changes and shaping short-term responses to environmental fluctuations.

## Supplementary Material

Rubio_Portillo_Supplementary_Figures_JUL25_ycaf157

Rubio-Portillo_Suppl_TABLES_ycaf157

Rubio_Portillo_Supplementary_methods_ycaf157

## Data Availability

Raw sequencing data, MAGs and vOTUs are available under the BioProject PRJNA1232376.
