# Correction to: Short-term virus-host interactions and functional dynamics in recently deglaciated Antarctic tundra soils

**DOI:** 10.1093/ismeco/ycag245

**Published:** 2026-09-07

This is a correction to: Esther Rubio-Portillo, Rebeca Arias-Real, Esther Rodríguez-Pérez, Lluis Bañeras, Josefa Antón, Asunción de los Ríos, Short-term virus-host interactions and functional dynamics in recently deglaciated Antarctic tundra soils, *ISME Communications*, Volume 5, Issue 1, January 2025, ycaf157, https://doi.org/10.1093/ismeco/ycaf157

In the originally published version of this manuscript, the supplementary methods file was erroneously omitted.

This has now been added to the ‘Supplementary Data’ section.

