# Supplementary material for: Short-term virus-host interactions and functional dynamics in recently deglaciated Antarctic tundra soils
Source: ISME Commun. 2025 Sep 9;5(1):ycaf157. doi: 10.1093/ismeco/ycaf157 (PMC12507030; doi:10.1093/ismeco/ycaf157)
Supplement: Rubio_Portillo_Supplementary_Figures_JUL25_ycaf157 [file rubio_portillo_supplementary_figures_jul25_ycaf157.pdf]

A)

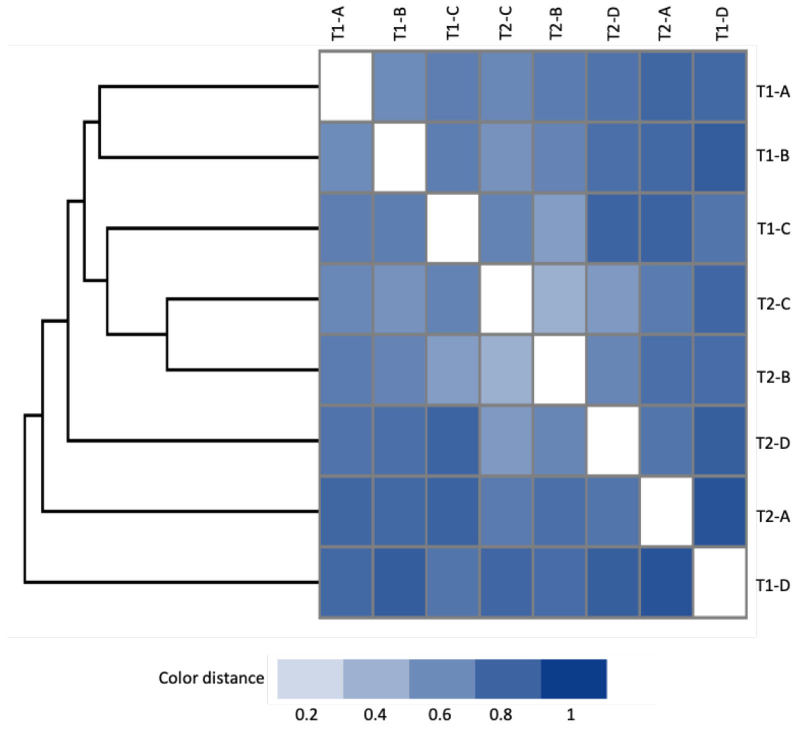

B)

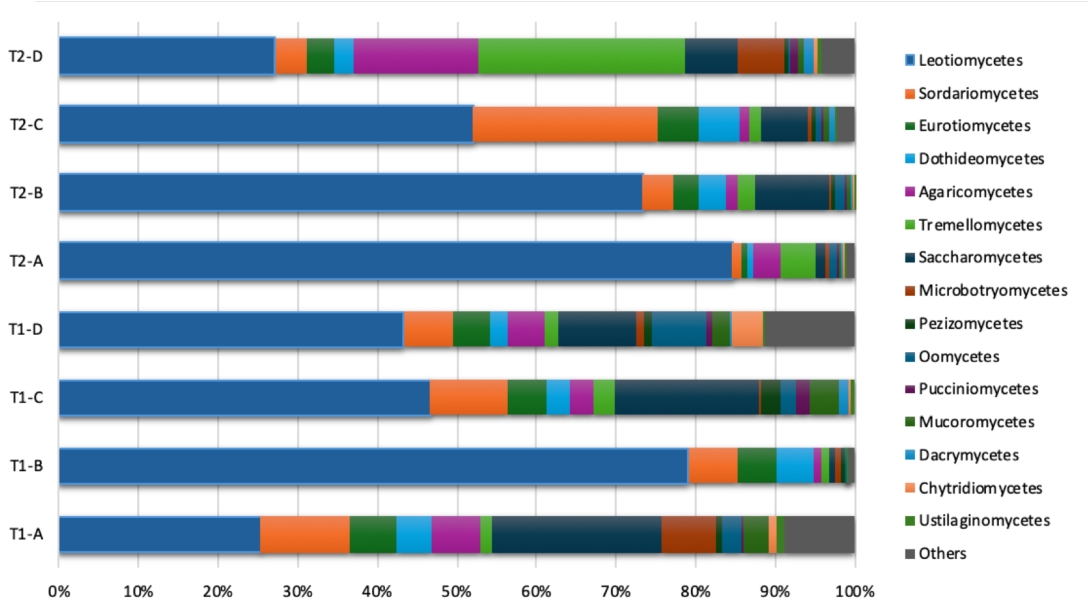

Fig. S1 A) Metagenomic clustering of metagenomes using Mash Comparison using raw sequencing data. Heat maps illustrate the pairwise similarity between samples, scaled between 0 (white) and 100 (blue). B) Relative abundance of fungal orders identified in reads classified as fungi within each metagenome.

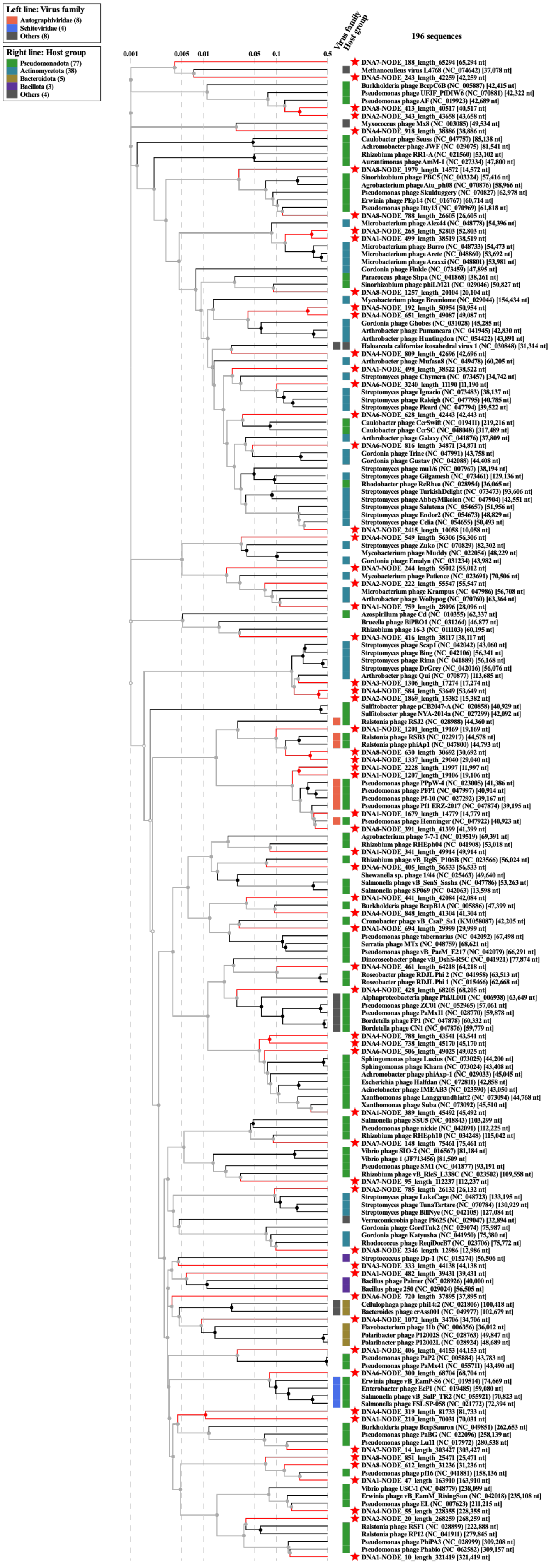

Fig. S2 Characterization of the viral contigs identified as lytic viruses. Proteomic tree calculated with VIPTREE of the lytic vOTUs and databases viruses. Viral OTUs are labeled as in SDS6. Stars note novel vOTUs (DNA1=T1-A; DNA2=T1-C; DNA3=T1-B; DNA4=T1-D; DNA5=T2-A; DNA6=T2-C; DNA7=T2-B; DNA8=T2-D)

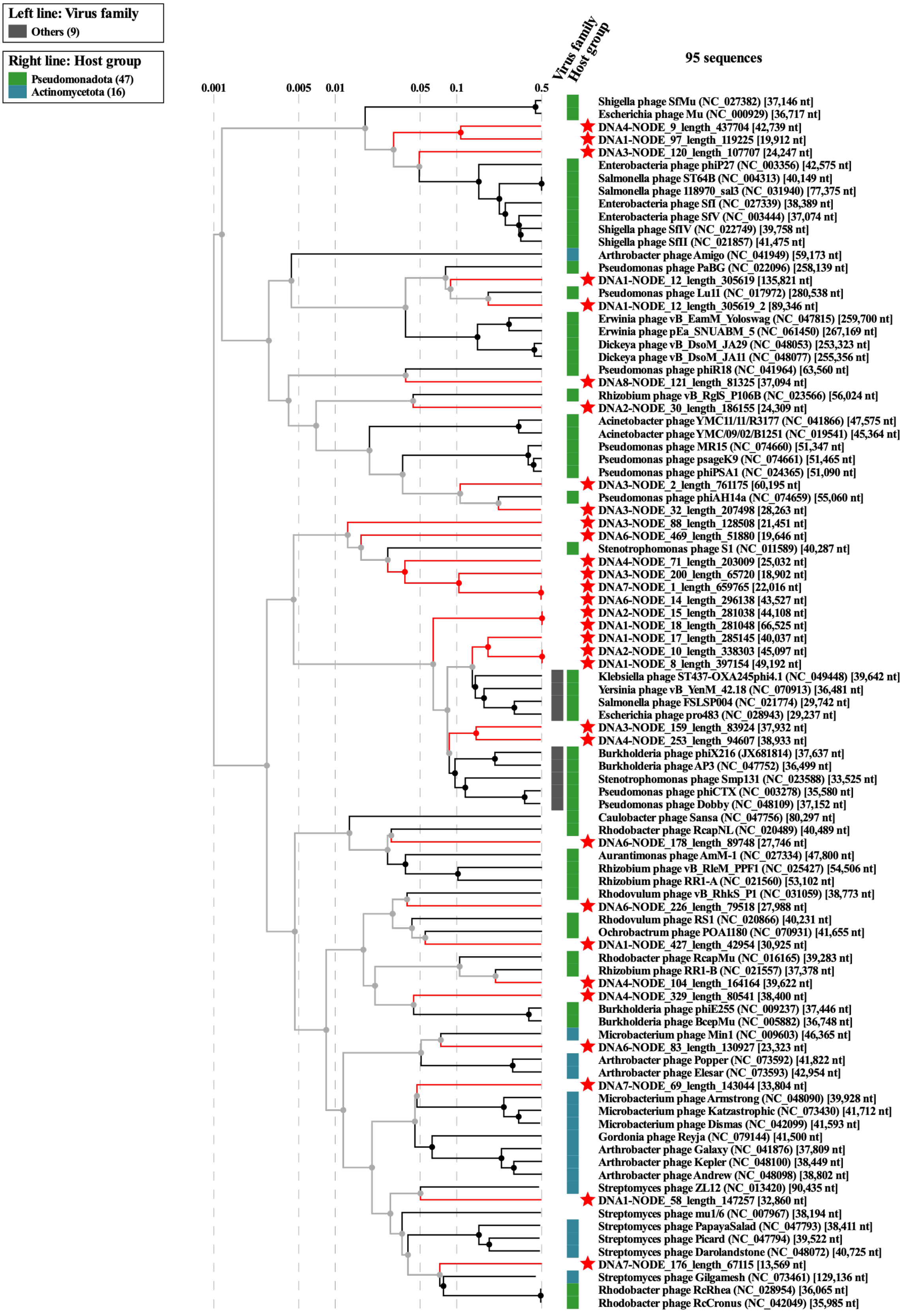

Fig. S3 Characterization of the viral contigs identified as prophages. Proteomic tree calculated with VIPTREE of the prophage vOTUs and viruses in databases. Viral OTUs are labeled as in SDS6. Stars note novel vOTUs (DNA1=T1-A; DNA2=T1-C; DNA3=T1-B; DNA4=T1-D; DNA5=T2-A; DNA6=T2-C; DNA7=T2-B; DNA8=T2-D).

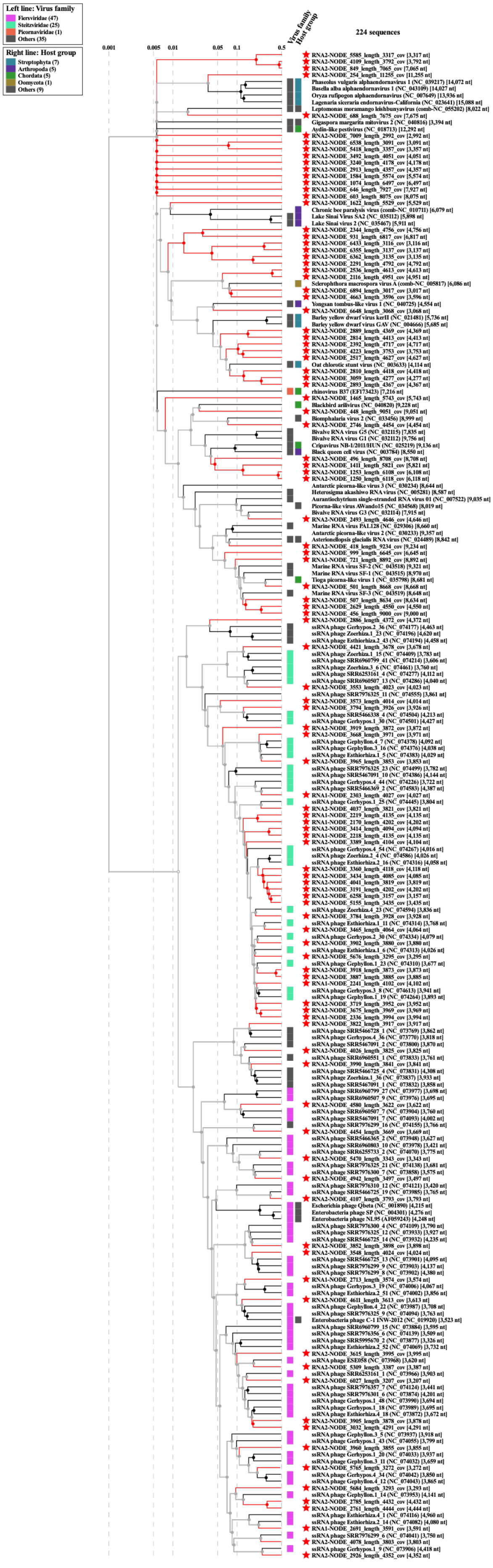

Fig. S4 Proteomic tree calculated with VIPTREE of the RNA vOTUs detected in the analyzed samples. Viral OTUs are labeled as in SDS6. Stars note novel vOTUs (DNA1=T1-A; DNA2=T1-C; DNA3=T1-B; DNA4=T1-D; DNA5=T2-A; DNA6=T2-C; DNA7=T2-B; DNA8=T2-D)

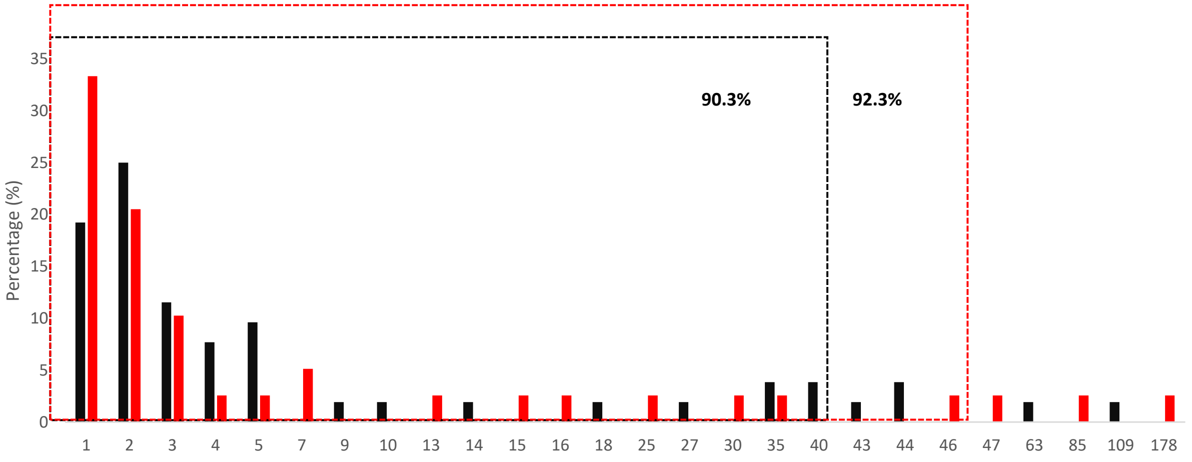

Fig S5. The distribution of CRISPR array sizes in Antartic soil metagenomes. Spacers for the T1 and T2 metagenomes are colored as red and black, respectively. Array size distributions are expressed as percentage of total number of arrays detected for each exposure time. Cutoffs are indicated by black and red dashed lines for both exposure times and represent the size range containing the majority (>90%) of the array population.
