# Supplementary material for: Short-term virus-host interactions and functional dynamics in recently deglaciated Antarctic tundra soils
Source: ISME Commun. 2025 Sep 9;5(1):ycaf157. doi: 10.1093/ismeco/ycaf157 (PMC12507030; doi:10.1093/ismeco/ycaf157)
Supplement: Rubio-Portillo_Suppl_TABLES_ycaf157 [file rubio-portillo_suppl_tables_ycaf157.pdf]

Table S1. GPS coordinates and soil attributes of the samples used in this study, including carbon content (C), nitrogen content (N), carbon and nitrogen ratio (C/N), organic matter content (OM) and Exposure Time (ET) measured in years since glacier retreat.

Sample	GPS Coordinates	N (g/100g)	C (g/100g)	C/N	OM (g/100g)	ET (years)
T1-A	62º 41.946´ S<br>60º 25.172´ W	0.012	0.16	12.828	0.3	11
T1-B	62º 41.955´ S<br>60º 25.147´ W	0.01	0.322	31.633	0.305	11
T1-C	62º 41.946´S<br>60º 25.172´ W	0.014	0.099	7.322	0.295	11
T1-D	62º 41.955´ S<br>60º 25.147´ W	0.01	0.052	5.117	0.251	11
T2-A	62º 41.992´ S<br>60º 25.123´ W	0.02	0.126	6.411	0.542	18
T2-B	62º 41.993´ S<br>60º 25.110´ W	0.08	0.081	9.647	0.253	18
T2-C	62º 41.992´ S<br>60º 25.123´ W	0.002	0.073	40.376	0.208	18
T2-D	62º 41.993´ S<br>60º 25.110´ W	0.014	0.055	3.807	0.246	18

Table S2. Marker genes involved in each step of carbon, nitrogen and sulfur cycles used in the functional characterization of the metagenomes and MAGs.

Cycle	Step	Marker gene
**Carbon**	Aerobic Carbon fixation	Phosphoribulokinase (*prkB*)<br>RuBisCO large chain (*rbcL*)<br>RuBisCO small chain (*rbcS*)
	Anaerobic Carbon fixation	ATP citrate lyase (*ACLY*)
	Aerobic methane oxidation	methane monooxygenase subunit A (*pmoA*)
	Aerobic respiration	cytochrome c oxidase subunit I (*coxI*)<br>cytochrome c oxidase subunit II (*coxII*)
	CO oxidation	CO dehydrogenase small subunit (*coxS*)
	Fermentation	L-lactate dehydrogenase (*LDH*)
	Methanogenesis	coenzyme M methyl reductase beta subunit (*mcrB*)
**Nitrogen**	Ammonification	formate-dependent nitrite reductase periplasmic cytochrome c552 (*nrfA*)<br>nitrite reductase (NADH) large subunit (*nirB*)<br>nitrite reductase (NADH) small subunit (*nirD*)<br>ferredoxin-nitrite reductase (*nirA*)
	Denitrification	nitrous oxide reductase (*nosZ*, *nosL*)<br>nitric-oxide reductase (*norB*)<br>nitrite reductase (NO-forming) (*nirK*)<br>periplasmic nitrate reductase (*napA*)
	Nitrification	ammonia monooxygenase subunit A (*amoA*)
	Nitrogen fixation	nitrogenase iron protein (*nifH*)
**Sulfur**	Assimilatory sulfate reduction	sulfate adenylyltransferase (*sat*)
	Dissimilatory sulfate reduction and sulfide oxidation	adenylylsulfate reductase subunit A (*aprA*)<br>sulfite reductase (*dsrA*)
	Sulfur mineralization	sulfite reductase (ferredoxin) (*sir*)
**Phototrophy**	Oxygenic phototrophy	photosystem I core protein (*psaA*)
	Anoxygenic phototrophy	Photosynthetic reaction centre M (*pufM*)
	Bacteriorhodopsin-like protein	Retinal-binding proteins (*Bac_rhodopsin*)

Table S3: Final competing models. Grey cells indicate the variables (i.e., different abiotic soil attributes and/or exposure time) included in a particular model.

	Carbon content	Nitrogen content	Organic matter content	C/N ratio	Exposure time	pH
Mod 1	■				■	
Mod 2		■			■	
Mod 3			■		■	
Mod 4				■	■	
Mod 5	■		■		■	
Mod 6	■			■	■	
Mod 7	■		■	■	■	
Mod 8			■	■	■	
Mod 9	■					
Mod 10		■				
Mod 11			■			
Mod 12				■		
Mod 13					■	
Mod 14						■
Mod 15	■				■	■
Mod 16		■			■	■
Mod 17			■		■	■
Mod 18				■	■	■
Mod 19	■		■		■	■
Mod 20	■			■	■	■
Mod 21	■		■	■	■	■
Mod 22			■	■	■	■

Table S4: Number of gene copies per unit gram of soil (ww) of the bacterial and archaeal 16S rRNA genes in samples of the T1 sampling area.

Sample	GPS Coordinates	q16S rRNA Bacteria (gene copies/g ww soil)	q16S rRNA Archaea (gene copies/g ww soil)
T1-AM1	62º 41,978´ S 60º 25,163´ W	$6.48\ 10^{7}$	$2.09\ 10^{7}$
T1AM2	62º 41,979´ S 60º 25,160´ W	$6.36\ 10^{10}$	$2.46\ 10^{7}$
T1-AM3	62º 41,979´S 60º 25,157´ W	$2.29\ 10^{10}$	$1.80\ 10^{7}$
T1-AM4	62º 41,978´ S 60º 25,164´ W	$4.44\ 10^{9}$	$6.06\ 10^{6}$
T1-AM5	62º 41,978´ S 60º 25,163´ W	$1.19\ 10^{10}$	$2.93\ 10^{7}$
T1-AD1	62º 41,978´ S 60º 25,163´ W	$2.30\ 10^{9}$	$2.00\ 10^{6}$
T1-AD2	62º 41,978´ S 60º 25,159´ W	$1.11\ 10^{10}$	$1.28\ 10^{7}$
T1-AD3	62º 41,985´ S 60º 25,159´ W	$4.44\ 10^{8}$	$9.36\ 10^{5}$
T1-AD4	62º 41,983´ S 60º 25,167´ W	$1.01\ 10^{10}$	$3.96\ 10^{7}$
T1-AD5	62º 41,981´ S 60º 25,166´ W	$3.84\ 10^{10}$	$1.07\ 10^{7}$

Table S5: Results of the indicator species analysis (IndVal), including the maximum indicator value (IV), its significance for each taxon, and the corresponding time exposure.

Taxa (Phylum)	Exposure time	iv	pval
Aenigmarchaeota	T1	0.84	0.023
Caldisericota	T1	0.81	0.021
Evosea	T1	0.74	0.038
Fermentibacteriota	T1	0.83	0.04
Fusobacteriota	T1	0.89	0.026
Iainarchaeota	T1	0.74	0.049
Margulisbacteria	T1	0.76	0.03
