# Supplementary material for: Short-term virus-host interactions and functional dynamics in recently deglaciated Antarctic tundra soils
Source: ISME Commun. 2025 Sep 9;5(1):ycaf157. doi: 10.1093/ismeco/ycaf157 (PMC12507030; doi:10.1093/ismeco/ycaf157)
Supplement: Supplementary file 3 [file rubio_portillo_supplementary_methods_ycaf157.pdf]

Supplementary Methods

TITLE: Short-term virus-host interactions and functional dynamics in recently deglaciated Antarctic tundra soils

Running Title: Short-term microbial dynamics in glacier forefields

AUTHORS: Esther Rubio-Portillo[1], Rebeca Arias-Real[2], Esther Rodríguez-Pérez[2], Lluis Bañeras[3], Josefa Antón[1]*, Asunción de los Ríos[2]*

AUTHOR AFFILIATIONS: [1]Department of Physiology, Genetics and Microbiology, University of Alicante, Alicante, Spain. [2]Department of Biogeochemistry and Microbial Ecology, Museo Nacional de Ciencias Naturales-CSIC, Spain. [3]Group of Molecular Microbial Ecology, Institute of Aquatic Ecology, University of Girona, Girona, Spain.

***CORRESPONDANCE:**

Asunción de los Ríos: arios@mncn.csic.es

Josefa Antón: anton@ua.es

Supplementary Methods

Comparative metabolic profile of the metagenomes. Protein-coding genes from contigs ≥ 1 kb were used to determine the metabolic profile of each sample using profile hidden Markov models (HMMs) in the *HMMER* package [1]. To construct the HMM model, a profile HMM was generated from an alignment of representative carbon, nitrogen and sulfur marker genes (Table S2) previously published for Antarctic glaciers [2]. Subsequent gene annotation was performed using *hmmsearch* [1] against each of the seven profiles subsets. The relative abundance of each gene was calculated by normalizing its abundance to the metagenome size.

MAG retrieval and analysis. Contigs larger than 5 kb were selected for metagenome binning using *MetaBAT* [3] and *MaxBin 2.0* [4] with final optimized bins obtained using *DAS Tool* 1.0 [5]. Genome completeness and contamination levels in Metagenome-Assembled Genomes (MAGs, post curation bins) were assessed with *Anvi'o* v.2.1.0 [6]. Following the standards suggested by [7], bins were considered high quality when completeness exceeded 80% and contamination remained below 5%. Functional annotation, which also provided taxonomy information, was performed by BLASTp comparisons of the predicted ORFs against the NCBI nr database. Bins were manually checked for consistent coverage and taxonomy coherence across contigs. Sixteen rRNA gene sequences were extracted from each Bin using *RNAscan* software [8] and compared against the SILVA-138-S database [9]. High-quality MAGs were further analyzed using fast pairwise comparison via the Mash algorithm [10] and clustered at 95% average nucleotide identity (ANI). After dereplication step, taxonomic classification of MAGs was assigned using the GTDB-Tk pipeline, which assigns genomes to GTDB reference database based on marker gene sets [11]. The metabolic potential for MAGs was reconstructed by functionally annotating predicted proteins using *BlastKOALA* [12]. The average amino acid identity (AAI) was calculated using *CompareM* v.0.1.0 (https://github.com/dparks1134/ CompareM).

To track the relative abundance of each MAG across samples, metagenomic short reads were mapped to MAG contigs using *BBMap* [13], filtering for single best alignments length with a minimum 90% query cover and 95% nucleotide identity (ANIr). To reduce biases from highly conserved regions and contig edges, abundance was estimated using

the 80% central truncated average sequencing depth of all bases (TAD80) as described by [14]. MAG abundance in each metagenomic dataset (as a percentage of the total community) was calculated as the quotient of the MAG's TAD80 value and the estimated genome equivalents (GE) from *MicrobeCensus* [15]. Reads corresponding to 16S rRNA genes were retrieved from metagenomes using *SortMeRNA* [16]. Antiviral defense systems within MAGs were identified using the *Defense Finder* software [17]. Antibiotic resistance genes (ARGs) were annotated by alignment against the Comprehensive Antibiotic Resistance Database (CARD) using RGI (v5.1.1).

Viral sequence analyses. We have considered as viral those metagenomic contigs ≥ 10 kb which were identified as such using at least three of the following four methods: (i) contigs included in 1 or 2 *VIRSorter* v1.03 categories [18], (ii) contigs that were identified as phage with complete circular or high quality draft using *VIBRANT* [19], (iii) contigs that were taxonomically classified as viruses using *Kraken* v0.10.5 and (iv) contigs that were taxonomically classified as viruses using kaiju [20]. Additionally, contigs ≥ 10 kb were identified as prophage using at least two of these three methods: (i) contigs included in 1 or 2 *VIRSorter* v1.03 categories [18], (ii) contigs that were identified as lysogenic with complete circular or high-quality draft using *VIBRANT* [19] and (iii) contigs annotated as intact o questionable prophages using *PHASTER* [21]. Contigs identified as viral, with at least 8% of their predicted genes annotated as viral genes via DIAMOND *BLASTp* against the NCBI nr database with an e-value <0.00001, were considered bona fide lytic viral genomes. To group viral sequences into viral operational taxonomic units (vOTUs), cdhit was used with a 95% average nucleotide identity and 80% alignment fraction threshold for the smallest contig using *Cd-hit* (cdhit-est -c 0.9 –n 8) [22] and removed using *FastA.filter.pl* [23]. *In silico* virus–host prediction was based on the best viral hit from DIAMOND *BLASTx* against the NCBI nr database, CRISPR systems identification and *PHISDetector* [24].

Viral contig comparisons were conducted using *vConTACT2* 0.9.09, with Prokaryotic Viral RefSeq version 201 (with ICTV and NCBI taxonomies), and connections with scores > 1 were considered. These networks were visualized using *Cytoscape* [25]. Further *in silico* virus–host prediction was based on the best viral hit by DIAMOND *BLASTx* against the NCBI nr database and *PHISDetector* [26]. Whole viral genome comparisons (in

nucleotides) were performed using VIRIDIC [27], and viral proteomic trees, including published viral genomes, were built using *VIPtree* [28].

qPCR analyses. Prior to the qPCR analysis, DNA samples were diluted to an approximate total concentration (0.1 to 0.05 ng/µL), if necessary. Inhibition assays were performed to detect any compounds that might affect amplification efficiency, especially in non-diluted samples. A known amount of plasmid containing no inserts was used as a standard for inhibition tests and added to DNA extracts at 10^5 copies/µL. qPCR reactions were performed using primers targeting plasmid sequences and the results were compared between reactions with plasmid only and those with a combination of plasmid plus sample. No inhibition was considered when the difference in the quantification between the two reactions was less than 10%. Higher dilutions were used if inhibitory effects were observed in any sample. All reactions were performed on a Lightcycler 96 Real-Time PCR system, using the LightCycler® 480 SYBR Green I Master (Roche Life Science, Basel, Switzerland). Each reaction had a final volume of 20µL containing 1×LightCycler ® 480 SYBR Green I Master, 35-40 ng of DNA, and 1µM of each primer. For bacteria 16S rRNA, primers 341F and 534R were used [29], while for archaeal 16S rRNA, primers Arch364aF and ArchA934b were used [30]. PCR conditions were as following. For 16S rRNA bacteria, a first denaturation step at 95ºC for 10 min followed by 35 cycles at 95ºC for 10 seconds, 60ºC for 10 seconds and 72ºC for 30 seconds. For archaeal 16S rRNA, the same cycling conditions were used except for the annealing step set at 66ºC for 30 seconds. For the two molecular markers, fluorescence was recorded as a single point at the end of the extension step.

Data analysis.

To reduce distribution skewness before analysis, the C/N ratio was square-root transformed, and organic matter content was log-transformed. All abiotic soil attributes were subsequently z-standardized (mean = 0, SD = 1). We then built twenty-two linear models (LMs) for each community structure metric and metabolic gene, including different combinations of time and abiotic soil attributes as predictors. Only models with variance inflation factors (VIF) < 2 were included, indicating acceptable levels of multicollinearity [31]. Models containing both nitrogen content and carbon/organic matter contents and/or the C/N ratio were excluded due to high collinearity (Table S3).

Second, we applied a multimodel inference approach [32,33] using the model.sel function from the MuMIn R package to estimate regression coefficients, statistical support, and the importance of time and abiotic soil predictors [34]. Based on the second-order Akaike Information Criterion (AICc), we ranked the models and retained those with $\Delta AICc \leq 2$ from the best model. To evaluate the explanatory power and contribution of individual predictors, we calculated both total and partitioned explained variance (R^2). For each response variable, the partitioned variance explained by each predictor was averaged across the top models using Akaike weights [35,36], reflecting the relative support for each variable across plausible models. All models were checked for linear model assumptions, including normality and homoscedasticity of residuals [31]. All statistical analyses were performed using R statistical software version 3.4.1 (R Development Core Team 2011).

To identify indicator taxa for the two exposure times analyzed (T1 and T2), we performed an indicator species analysis (IndVal) at the phylum level, as described by [37], using the R packages *vegan*, *labdsv*, and *ade4*. This analysis computes an indicator value index (IV) for each taxon based on its presence or absence in the taxa composition matrix and corresponding exposure time. The IV calculation incorporates both specificity (maximized when a taxon occurs exclusively at one time) and fidelity (maximized when a taxon is present at both exposure times).

Supplementary References

1. Eddy SR. Accelerated profile HMM searches. *PLoS Comput Biol* 2011;7:e1002195.
2. Li Y, Cha QQ, Dang YR, Chen XL, Wang M, McMinn A. Reconstruction of the functional ecosystem in the high light, low temperature union glacier region, Antarctica. *Front Microbiol* 2019;10:2408.
3. Kieft K, Zhou Z, Anantharaman K. VIBRANT: automated recovery, annotation and curation of microbial viruses, and evaluation of viral community function from genomic sequences. *Microbiome* 2020;8:1–23.
4. Wu YW, Tang YH, Tringe SG, Simmons BA, Singer SW. 2014. MaxBin: an automated binning method to recover individual genomes from metagenomes using an expectation-maximization algorithm. *Microbiome* 2014;2:26.

5. Sieber CM, Probst AJ, Sharrar A, Thomas BC, Hess M, Tringe SG, Banfield JF. Recovery of genomes from metagenomes via a dereplication, aggregation and scoring strategy. *Nat Microbiol* 2018;3:836–43.
6. Eren AM, Esen ÖC, Quince C, Vineis JH, Morrison HG, Sogin ML, Delmont. 2015. Anvi'o: an advanced analysis and visualization platform for 'omics data. *PeerJ* 2015;3:e1319.
7. Konstantinidis KT, Rosselló-Móra R, Amann R. Uncultivated microbes in need of their own taxonomy. *ISME J* 2017;11:2399–2406.
8. Lagesen K, Hallin P, Rodland EA, Staerfeldt HH, Rognes T, Ussery DW. RNAmmer: consistent and rapid annotation of ribosomal RNA genes. *Nucleic Acids Res* 2007;35:3100–08.
9. Quast C, Pruesse E, Yilmaz P, Gerken J, Schweer T, Yarza P, Peplies J, Glöckner FO. The SILVA ribosomal RNA gene database project: improved data processing and web-based tools. *Nucleic Acids Res* 2013;41:D590–96.
10. Ondov BD, Treangen T J, Melsted P, Mallonee AB, Bergman N, Koren S, Phillippy AM. Mash: fast genome and metagenome distance estimation using MinHash. *Genome Biol* 2016;17:1–14.
11. Chaumeil PA, Mussig AJ, Hugenholtz P, Parks DH. GTDB-Tk: a toolkit to classify genomes with the Genome Taxonomy Database. *Bioinformatics* 2020;36:1925–27.
12. Kanehisa M, Sato Y, Morishima K. BlastKOALA and GhostKOALA: KEGG Tools for Functional Characterization of Genome and Metagenome Sequences. *J Mol Biol.* 2016; 22:726-731.
13. Bushnell B. BBMap: a fast, accurate, splice-aware aligner (No. LBNL-7065E). Lawrence Berkeley National Lab., Berkeley, CA (United States), 2024.
14. Rodriguez RLM, Tsementzi D, Luo C, Konstantinidis KT. Iterative subtractive binning of freshwater chronoseries metagenomes identifies over 400 novel species and their ecologic preferences. *Environ Microbiol* 2020;22:3394–3412.
15. Nayfach S, Pollard KS. Average genome size estimation improves comparative metagenomics and sheds light on the functional ecology of the human microbiome. *Genome Biol* 2015;16:51
16. Kopylova E, Noe L, Touzet H. SortMeRNA: fast and accurate filtering of ribosomal RNAs in metatranscriptomic data. *Bioinformatics* 2012;28:3211–17.

17. Tesson F., Planel R., Egorov A.A., Georjon H., Vaysset H., Brancotte B., Néron B., Mordret E., Atkinson G.C., Bernheim A.et al. . A comprehensive resource for exploring antiphage defense: DefenseFinder webservice, Wiki and databases. *Peer Community J* 2024; 4:91.

18. Roux S, Enault F, Hurwitz BL, Sullivan MB. VirSorter: mining viral signal from microbial genomic data. *PeerJ* 2015;3:e985.
19. Kieft K, Zhou Z, Anantharaman K. VIBRANT: automated recovery, annotation and curation of microbial viruses, and evaluation of viral community function from genomic sequences. *Microbiome* 2020;8:1–23.
20. Menzel P, Ng KL, Krogh A. Fast and sensitive taxonomic classification for metagenomics with Kaiju. *Nat Commun* 2016;7:11257–59.
21. Arndt D, Grant JR, Marcu A, Sajed T, Pon A, Liang Y, Wishart DS. (2016). PHASTER: a better, faster version of the PHAST phage search tool. *Nucleic Acids Res* 2016;44:W16–21.
22. Li W, Godzik A. Cd-hit: a fast program for clustering and comparing large sets of protein or nucleotide sequences. *Bioinformatics* 2006;22:1658–9.
23. Rodriguez RLM, Konstantinidis KT. The enveomics collection: a toolbox for specialized analyses of microbial genomes and metagenomes. *PeerJ Preprints* 2016;4:e1900v1.
24. Zhang F, Zhou F, Gan R, Ren C, Jia Y, Yu L, Huang Z. PHISDetector: a tool to detect diverse in silico phage-host interaction signals for virome studies. *bioRXiv* 2019; 661074.
25. Shannon P, Markiel A, Ozier O, Baliga NS, Wang JT, Ramage D, Amin N, Schwikowski B, Ideker T. *Cytoscape*: a software environment for integrated models of biomo-lecular interaction networks. *Genome Res* 2003;13:2498–504.
26. Zhou F, Gan R, Zhang F, Ren C, Yu L, Si Y. PHISDetector: A tool to detect diverse In Silico Phage–host interaction signals for virome studies. *Genomics Proteomics Bioinformatics* 2022;20:508–23.
27. Moraru, C., Varsani, A., and Kropinski, A.M. (2020) VIRIDIC-A novel tool to calculate the intergenomic similarities of prokaryote-infecting viruses. *Viruses* **12**: 1268.
28. Nishimura, Y., Yoshida, T., Kuronishi, M., Uehara, H., Ogata, H., and Goto, SViPTree:

The viral proteomic tree server. *Bioinformatics* 2017; 33:2379–80.

29. Henry S, Baudoin E, López-Gutiérrez JC, Martin-Laurent F, Brauman A, Philippot L. Quantification of denitrifying bacteria in soils by nirK gene targeted real-time PCR. *J Microbiol Methods* 2004;59:327–35.
30. Kemnitz, D., S. Kolb, Conrad R. High abundance of Crenarchaeota in a temperate acidic forest soil. *FEMS Microb Ecol* 2007; 60:442-448.
31. Zuur AF, Ieno EN, Elphick CS. A protocol for data exploration to avoid common statistical problems. *Methods Ecol Evol* 2010;1:3–14.
32. Burnham KP, Anderson DR, Huyvaert KP. (2011). AIC model selection and multimodel inference in behavioral ecology: some background, observations, and comparisons. *Behav Ecol Sociobiol* 2011;65:23–35.
33. Grueber CE, Nakagawa S, Laws, RJ, Jamieson IG. Multimodel inference in ecology and evolution: challenges and solutions. *J evol biol*, 2011; 24, 699-711.
34. Barton K. MuMIn: multi-model inference. R package version 1.43. 17. 2020
35. Hoffman GE, Schadt, EE. VariancePartition: interpreting drivers of variation in complex gene expression studies. *BMC bioinformatics* 2016;17:1–13.
36. Giam X., Olden JD. Quantifying variable importance in a multimodel inference framework. Meth Ecol Evol, 2016; 7, 388-397.
37. Dufrene, M. and Legendre, P. Species assemblages and indicator species: the need for a flexible asymmetrical approach. *Ecol Monogr* 1997.67:345-366.
